# Picroside II Attenuates Airway Inflammation by Downregulating the Transcription Factor GATA3 and Th2-Related Cytokines in a Mouse Model of HDM-Induced Allergic Asthma

**DOI:** 10.1371/journal.pone.0167098

**Published:** 2016-11-21

**Authors:** Jin Choi, Bo Kyong Choi, Jin seok Kim, Jae-Won Lee, Hyun Ah Park, Hyung Won Ryu, Su Ui Lee, Kwang Woo Hwang, Won-Kee Yun, Hyoung-Chin Kim, Kyung-Seop Ahn, Sei-Ryang Oh, Hyun-Jun Lee

**Affiliations:** 1 Natural Medicine Research Center, Korea Research Institute of Bioscience and Biotechnology (KRIBB), Cheongju, Chungbuk, Republic of Korea; 2 College of Pharmacy, Chung-Ang University, Seoul, Republic of Korea; 3 Laboratory Animal Resource Center, Korea Research Institute of Bioscience and Biotechnology (KRIBB), Cheongju, Chungbuk, Republic of Korea; Mie University Graduate School of Medicine, JAPAN

## Abstract

Picroside II isolated from *Pseudolysimachion rotundum* var. *subintegrum* has been used as traditional medicine to treat inflammatory diseases. In this study, we assessed whether picroside II has inhibitory effects on airway inflammation in a mouse model of house dust mite (HDM)-induced asthma. In the HDM-induced asthmatic model, picroside II significantly reduced inflammatory cell counts in the bronchoalveolar lavage fluid (BALF), the levels of total immunoglobulin (Ig) E and HDM-specific IgE and IgG1 in serum, airway inflammation, and mucus hypersecretion in the lung tissues. ELISA analysis showed that picroside II down-regulated the levels of Th2-related cytokines (including IL-4, IL-5, and IL-13) and asthma-related mediators, but it up-regulated Th1-related cytokine, IFNγ in BALF. Picroside II also inhibited the expression of Th2 type cytokine genes and the transcription factor GATA3 in the lung tissues of HDM-induced mice. Finally, we demonstrated that picroside II significantly decreased the expression of GATA3 and Th2 cytokines in developing Th2 cells, consistent with *in vivo* results. Taken together, these results indicate that picroside II has protective effects on allergic asthma by reducing GATA3 expression and Th2 cytokine bias.

## Introduction

Allergic asthma is a chronic inflammatory disease characterized by infiltration of the airway wall with eosinophils and CD4+ T helper type 2 (Th2) cells, reversible airway obstruction, airway hyper-responsiveness (AHR), mucus hypersecretion and airway remodeling [1, 2]. The risk factors for developing asthma are a combination of genetic predisposition and environmental pollutions such as pollen, animal dander and mites [3–5]. Among many factors, house dust mite (HDM) is the most prevalent cause of allergic sensitization [4, 6], and a risk factor for persistent asthma in human subjects [7, 8].

Exposure to allergens causes differentiation of CD4+ T cells into effector T cells, Th1, Th2 and Th17 cells, which produce lineage-specific cytokines IFNγ, IL-4 and IL-17, respectively [9, 10]. The appropriate differentiation of these CD4+ T-cell subsets requires activation of transcription factors, including signal transducers and activators of transcription (STATs) [11]. STAT signals have been shown to be involved in the induction of subset-specific transcription factors: T-box expressed in T-cells (T-bet) for Th1; GATA-binding protein-3 (GATA3) for Th2; and retinoic acid receptor-related orphan receptor γt (RORγt) for Th17 cells [12–15]. GATA3 induces Th2 cell development by promoting Th2 cytokine expression, but simultaneously inhibits Th1 differentiation by inhibiting T-bet and thus plays a critical role in asthma [14, 16, 17]. Asthma is controlled by Th2 immune response, which leads to the high levels of IgE, airway eosinophilia, and mucus production [18]. Therefore, excessive cytokines IL-4, IL-5, and IL-13 produced by Th2 cells are significant risk factors in asthma pathogenesis [19]. Thus, suppression of overproduced Th2 cytokines is important for the treatment of the disease.

Picroside II (PIC II), a catalpol derivative, is isolated from *Pseudolysimachion rotundum* var. *subintegrum* that has been used as traditional medicine to treat inflammatory diseases [20]. YPL-001 (drug substance) derived from the herb is currently on phase-2a clinical trials in Chronic Obstructive Pulmonary Disease (COPD). Picroside II has been shown to possess a broad range of pharmacological effects, including an anti-oxidant and anti-inflammation property [21–23]. In addition, picroside II is also known as major constituent in the rhizome of *Picrorhiza scrophulariiflora*, and often used in Asian traditional medicine for the treatment of a number of diseases [24]. However, picroside II-mediated anti-inflammatory effect in a mouse model of asthma has not been investigated.

In this study, we investigated the anti-inflammatory effects of the picroside II in a HDM-induced asthma mouse model as well as in developing Th2 cells. Picroside II attenuated Th2-driven allergic airway inflammation and suppressed GATA3 and Th2 cytokine production significantly *in vivo*. We further showed that picroside II inhibited GATA3-driven Th2 cytokines in developing Th2 cells. Therefore, we suggest that picroside II has potential for the treatment of HDM-induced asthma by reducing Th2-mediated airway inflammation.

## Materials and Methods

### Preparation of plants

Picroside II and YPL-001 (drug substance) was purified as described previously [25]. Picroside II purity was more than 99.5% as determined by ultra-performance liquid chromatography (S1 and S2 Figs). The drug substance of *P*. *rotundum* var. *subintegrum* was produced by the processing method described in ICH (International Conference on Harmonisation) and FDA (Food and Drug Administration) guidelines (Korean patent 10–1476095).

### Cell viability assay

T cells were cultured in 96-well plates at a density of 1 X 10^5^ cells/well. Picroside II extracts were added to each individual well and then incubated for 3 days. Cell viability was measured in triplicate using a Cell Counting Kit-8 (Dojindo Molecular Technologies, Rockville, ML) according to the manufacturer’s protocol. The optical density was measured at 570nm using a microplate reader (Tecan trading AG, Switzerland).

### Animals

Female BALB/c mice were purchased from the DBL (Eumseong, Korea) and housed in environmentally controlled pathogen-free conditions throughout the experiments. All experimental procedure were approved by the Institutional Animal Care and Use Committee of the Korea Research Institute of Bioscience and Biotechnology, and performed in compliance with the National Institute of Health Guidelines for the care and use of laboratory animals and the Korean National Animal Welfare Law.

### Allergen sensitization, challenge and treatment

**Fig 1:**
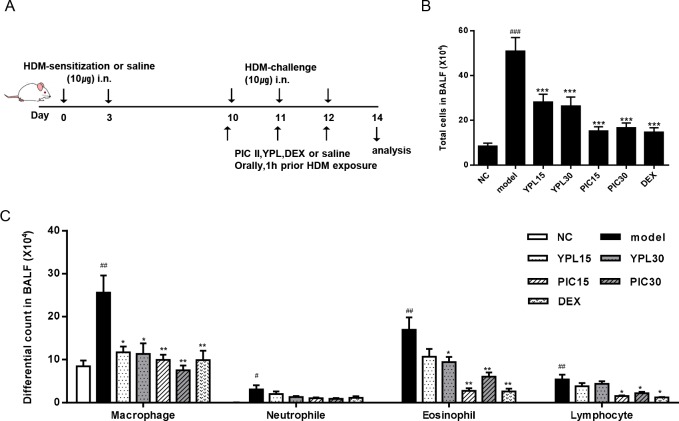
Effects of YPL-001(YPL) and picroside II (PIC II) on House dust mite (HDM)-induced BALF composition. (A) A timeline of allergen sensitization, exposure, and drug treatment in this study (DEX; dexamethasone, i.n; intranasal). The total cells (B) and differential cells (C) in BALF of mice were collected at 48h after the last HDM challenge, and quantified in DiffQuick-stained reagent. NC; normal control mice treated with saline only, model; HDM-sensitized/challenged mice, YPL 15 and 30; YPL-001 (15 and 30 ㎎/㎏) + HDM-sensitized/challenged mice, PIC 15 and 30; picroside II (15 and 30 ㎎/㎏)+HDM-sensitized/challenged mice, DEX; dexamethasone + HDM-sensitized/challenged mice. All data are representative of three independent experiments and represented as the mean ± SEM (n = 6 mice/group). ^#^p<0.05, ^##^p<0.01, and ^###^p<0.001, compared with normal control (NC); *p<0.05, **p<0.01, and ***p<0.001, compared with model group.

Mice were randomly divided into 5 groups and each consisted of 6 mice, namely normal control group (NC), model group (model), YPL-001 treated group (YPL, 15 and 30 ㎎/㎏) or picroside II treated group (PIC II,15 and 30 ㎎/㎏), and dexamethasone treated group (DEX, 1 ㎎/㎏). We induced asthma as previously described with some modification [26]. Briefly, the mice were sensitized to either purified HDM extract (10㎍ of protein in 50㎕ of saline; Greer Laboratories, Lenoir, NC) or saline (for normal control group) intranasally (i.n.) on days 0 and 3. Allergen challenge was done by intranasal treatment of HDM on three consecutive days (day 10–12) and sacrificed 48h after the last challenge (day 14) as shown in Fig 1A. To evaluate the protective effect, YPL-001 or picroside II were administered orally 1h before the HDM challenges for 3 days. As saline was used as vehicle, normal control group and model group were received only saline. Dexamethasone (1㎎/㎏) as a positive drug [27], was treated with the same procedure as for picroside II.

### Bronchoalveolar lavage fluid (BALF) collection and analysis

The BALF was performed by injecting ice-cold PBS (0.5ml) and withdrawing as much fluid as possible using a tracheal cannula with a 20G blunt needle. The procedure was repeated once for each mouse, and the BALF was pooled. BALF differential cell counts were performed on cytospin slides using Diff-Quik® staining reagent (IMEB Inc, Deerfield, IL) according to the manufacturer’s instructions. The supernatant was stored at -80°C until analysis for cytokines in BALF.

### Measurement of total IgE, HDM-specific IgE, and HDM-specific IgG1 by ELISA

Total serum IgE was assessed using OptEIA ELISA kits (BD Biosciences, San Diego, CA) according to the manufacturer’s instructions. An indirect ELISA method was used to assess the HDM-specific IgE and IgG1 levels in serum samples. Briefly, 96-well microtiter plates were coated overnight with 100㎍/mL HDM in PBS. The next day, 200㎕ of blocking solution (1% BSA in PBS) was added to the plate before the addition of serum samples that had been diluted 1:200 (for HDM-specific IgG1) in blocking buffer, or undiluted (for HDM-specific IgE) for 1h. Subsequently, 100㎕ of sample was added to the plate for 2 h, followed by biotin-rat-anti-mouse IgE or biotin-rat-anti-mouse IgG1 (2㎍/ml; BD Biosciences, San Diego, CA) for 1 h. To detect biotin-labeled IgE or IgG1, streptavidin-HRP (1:100, R&D Systems, Minneapolis, MN) was added to the plate for 30 min. Next, a tetramethylbenzidine substrate reagent set (1:1, BD Bioseicences) was added to detect levels of IgE or IgG1.

### Measurement of cytokine levels in BALF by ELISA

The levels of cytokines in BALF were analyzed with a commercially available specific OptEIA sandwich ELISA kit (BD Biosciences) by following the instructions of the manufacturer.

### Lung tissue histopathology

For histological analysis, lung tissues were excised intact and fixed for 24h in 4% (v/v) neutral buffered formalin. The tissues were embedded in paraffin and sectioned at 4-μm thickness. To estimate inflammation, lung sections were stained with a hematoxylin and eosin (H&E) solution (Sigma-Aldrich Inc, St. Louis, MO). Lung sections were also stained with periodic acid Schiff (PAS, IMEB, San Marcos, CA) to detect mucus production. Subsequently, the stained tissue was mounted and evaluated by microscopy.

### Quantitative real-time Reverse Transcription (RT)-PCR

Total RNA was extracted from homogenized lung tissues using TRIzol reagent (ambion, Carlsbad, CA) according to manufacturer’s protocol. The first strand cDNA was synthesized 1 ㎍ of total RNA and 1 μM Oligo-dT_18_ primer using Omniscript Reverse Transcriptase (Qiagen Inc, Valencia, CA). For real-time RT-PCR, the products were detected using the iQ SYBR Green supermix (Bio-Rad, Hercules, CA) using oligonucleotide sequences of PCR primers sets (Table 1). Thermal cycling and fluorescence detection were performed using the S1000 Thermal cycler real-time PCR system (Bio-Rad). Reactions run in a CFX96 Real-Time PCR System (Bio-Rad) using the following thermal conditions: an initial denaturation step at 95°C for 3min and 40 cycles of denaturation 95°C for 10s and annealing/extension at 55°C for 30s. The relative gene expression levels were evaluated by the ratio to *Gapdh* mRNA.

**Table 1:** Primer sequences for quantitative real-time PCR.

Genes	Sense (5' to 3')	Antisense (5' to 3')	Length (bp)
*Gapdh*	CCTGCACCACCAACTGCTTA	GTCTTCTGGGTGGCAGTGAT	109
*Ifng*	ATCTGGAGGAACTGGCAAAA	GCTGATGGCCTGATTGTCTT	108
*Il4*	AACGAGGTCACAGGAGAAGG	TTGGAAGCCCTACAGACGAG	107
*Il5*	ATCAAACTGTCCGTGGGGGT	TCTCCTCGCCACACTTCTCT	99
*Il13*	CCCTGGATTCCCTGACCAAC	CCAGGGATGGTCTCTCCTCA	211
*Muc5ac*	CCAGCAATCCCCTTTCCGAT	CCCTGCGGACAGTTGATCTT	292
*Il33*	AGAGATCCTTGCTTGGCAGT	AGCACCTGGTCTTGCTCTTG	199
*Mcp1*	CTTCTGGGCCTGCTGTTCA	CCAGCCTACTCATTGGGATCA	127
*Tbx21*	CTGGAGCTGGTTGGCCCGTG	GGACTCCGGCTGGAGGGAGG	166
*Gata3*	AGGCAACCACGTCCCGTCCT	TTTGCCGCCATCCAGCCAGG	135
*Il17*	CAGCAGCGATCATCCCTCAAAG	CAGGACCAGGATCTCTTGCTG	301

### Western blot analysis

The expressions of T-bet and GATA3 in lung tissues were detected by western blot. We homogenized the lung tissues in tissue protein extraction buffer (Thermo Fisher Scientific, Rockford, IL) in the presence of protease inhibitor cocktail to obtain extracts of lung proteins. The proteins were fractionated on 10% SDS-polyacrylamide gels. They were transferred to polyvinylidene fluoride membranes, and the membranes were incubated for 1h in 5% skim milk in TBS-T buffer (0.1 M Tris-HCl, pH 7.4, 0.9% NaCl, 0.05% Tween-20) to block non-specific binding and was then incubated with primary antibodies for overnight at 4°C. Antibody against GATA3, T-bet, GAPDH and secondary antibodies were obtained from Santa Cruz (Dallas, TX). The immunoblots were washed and incubated with appropriate secondary antibodies for 1 h. The blot was developed using an ECL kit (Thermo Fisher Scientific).

### *In vitro* Th cell differentiation and analysis

The splenocytes were isolated from sacrificed BALB/c mice. CD4+ T cells were isolated using anti-CD4 micro-beads according to the manufacturer’s instructions (Miltenyl Biotec., Auburn, CA). CD4+ T cells were stimulated with plate-bound anti-CD3 (0.5 ㎍/㎖) and anti-CD28 (1 ㎍/㎖). The cells were additionally treated with IL-2 (2 ng/ml) for the generation of Th0 or with IL-4 (10 ng/ml) and anti-IFNγ (5 ㎍/ml) for Th2. Three hours after initial culture, cells were treated with either vehicle or picroside II, and cultured for 3 days. Cell supernatants were used for ELISA, and cell pellets were harvested for real-time RT-PCR or western blot as described above.

SDS-PAGE was probed with antibodies against GATA3, phosphorylated STAT6 (Cell Signaling Technology, Danvers, MA) and STAT6 (Santa Cruz, Dallas, TX).

### Statistical analysis

Data were analyzed and graphed with GraphPad Prism software (version 6.07) and are presented as means ±SEMs. Statistical analysis was calculated using analysis of variance (ANOVA) followed by a multiple comparison test with Dunnet’s adjustment. *P* values of less than 0.05 were considered statistically significant.

## Results

### Effect of picroside II on the inflammatory cell count in the BALF

To investigate the protective effects of picroside II on airway inflammation, we used the HDM-induced asthma model. We first compared the effects of YPL-001 and picroside II on airway inflammation by counting inflammatory cells in BALF. As shown in Fig 1B and 1C, the numbers of total and differential inflammatory cells in BALF, including macrophage, neutrophils eosinophils, and lymphocytes, were markedly elevated in the HDM-treated model group compared to the normal controls (NC). Oral administration of picroside II in HDM-treated mice significantly reduced the total number of BALF inflammatory cells, specifically in the number of macrophage (*p*<0.01), eosinophils (*p*<0.01) and lymphocyte (*p*<0.05) compared to the HDM-treated model mice. Interestingly, picroside II showed better inhibitory effects than YPL-001. Moreover, the inhibitory effect of picroside II was comparable to those of dexamethasone. Thus, we focused onprotective effects of picroside II in further study.

### Effects of picroside II on airway inflammation in the lung tissues of HDM-challenged mice

**Fig 2:**
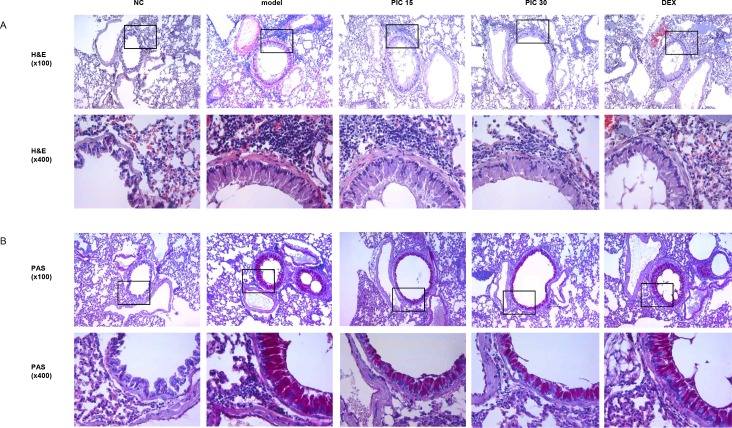
Effects of picroside II on airway inflammation and mucus secretion in HDM-induced asthma model. After the collection of BALF, lung tissue was fixed, sectioned at 4μm and stained with hematoxylin and eosin (H&E) (A) or periodic acid-Schiff (PAS) (B) solution (magnification 100x or 400x). NC; normal control mice treated with saline only, model; HDM-sensitized/challenged mice, PIC 15 and 30; picroside II (15 and 30 ㎎/㎏) + HDM-sensitized/challenged mice, DEX; dexamethasone + HDM-sensitized/challenged mice.

To estimate the anti-inflammatory effects of picroside II, histological inflammatory responses of lung tissues were assessed by H&E staining. Compared to the normal control mice, HDM-treatment caused inflammatory cell infiltration in the airway, including eosinophils, lymphocytes, neutrophils and macrophages. Treatment of picroside II clearly reduced the accumulation of inflammatory cells (Fig 2A). We also examined mucus metaplasia in PAS-stained lung sections. The results revealed marked accumulation of mucus secreting goblet cells in the epithelium of HDM-treated mice. In contrast, picroside II dramatically inhibited the number of PAS-stained goblet cells (Fig 2B).

### Effects of picroside II on the serum immunoglobulin responses

**Fig 3:**
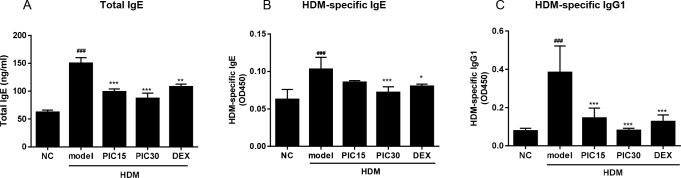
Effects of picroside II on total IgE, HDM-specific IgE and HDM-specific IgG1 in serum. Serum samples were collected 48h after the last HDM challenge. The levels of (A) total IgE, (B) HDM-specific IgE, and (C) HDM-specific IgG1 were measured using ELISA. NC; normal control mice treated with saline only, model; HDM-sensitized/challenged mice, PIC 15 and 30; picroside II (15 and 30 ㎎/㎏) + HDM-sensitized/challenged mice, DEX; dexamethasone + HDM-sensitized/challenged mice. All data are representative of three independent experiments and represented as the mean ± SEM (n = 6 mice/group). ^###^p<0.001, compared with normal control (NC); *p<0.05, **p<0.01, and ***p<0.001, compared with model group.

Allergen-specific IgE has been known as a key player in the pathophysiology of asthma [28]. Therefore, total IgE, HDM-specific IgE, and HDM-specific IgG1 levels in serum were measured by ELISA at 48h after the last HDM challenge. HDM-treated mice showed significantly higher serum levels of total IgE as well as HDM-specific IgE and IgG1 compared to the normal control (Fig 3). Administration of picroside II in HDM-treated mice significantly reduced serum total IgE, HDM-specific IgE, and HDM-specific IgG1 levels. The suppressive effects of picroside II were comparable, if not better than those of dexamethasone.

### Effects of picroside II on cytokine secretion in the BALF

**Fig 4:**
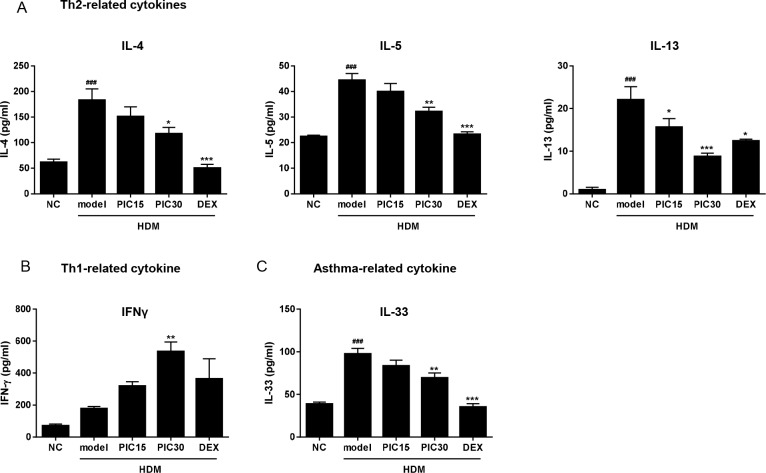
Effects of picroside II on cytokine levels in BALF. BALF samples were collected from mice 48h after the last HDM challenge. The levels of (A) Th2-related cytokines, (B) Th1-related cytokine, and (C) asthma-related cytokine were measured using ELISA. NC; normal control mice treated with saline only, model; HDM-sensitized/challenged mice, PIC 15 and 30; picroside II (15 and 30 ㎎/㎏) + HDM-sensitized/challenged mice, DEX; dexamethasone + HDM-sensitized/challenged mice. All data are representative of three independent experiments and represented as the mean ± SEM (n = 6 mice/group). ^###^p<0.001, compared with normal control (NC); *p<0.05, **p<0.01, and ***p<0.001, compared with model group.

An imbalance between the Th1 and Th2 responses contributes to the pathogenesis of allergic asthma [29]. To investigate the anti-asthmatic effects of picroside II, we determined the levels of inflammatory cytokines, such as Th1 cytokine (IFNγ), Th2 cytokines (IL-4, IL-5, and IL-13), and asthma related cytokine (IL-33) in the BALF using ELISA kit. ELISA showed that all the cytokine levels in model group mice were higher than those in normal group mice (Fig 4). Administration of picroside II significantly attenuated the Th2 related cytokines (including IL-4, IL-5 and IL-13) and IL-33. On the other hand, the level of IFN-γ was significantly elevated by picroside II in a dose-dependent manner. Dexamethasone suppressed all the cytokines measured, except IFN-γ. These results suggested that picroside II may affect Th1/Th2 balance in allergic asthma.

### Effects of picroside II on cytokine mRNAs levels

**Fig 5:**
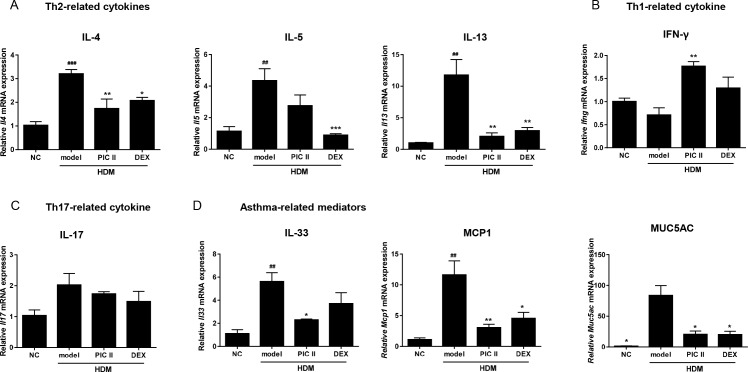
Effects of picroside II on mRNA expression of inflammatory cytokines/mediators in lung tissues. The mRNA levels of (A) Th2-related cytokines, (B) Th1-related cytokine, (C) Th17-related cytokine, and (D) asthma-related mediators, were determined by real-time RT-PCR. The data were normalized to *Gapdh* gene expression. NC; normal control mice treated with saline only, model; HDM-sensitized/challenged mice, PIC II; picroside II (30㎎/㎏) + HDM-sensitized/challenged mice, DEX; dexamethasone + HDM-sensitized/challenged mice. All data are representative of three independent experiments and represented as the mean ± SEM (n = 6mice/group). ^##^p<0.01 and ^###^p<0.001, compared with normal control (NC); *p<0.05, **p<0.01, and ***p<0.001, compared with model group.

Since ELISA in BALF showed that picroside II inhibits Th2-related cytokine secretion, we next examined the expression of transcripts for asthma associated cytokines and mediators from HDM-induced asthmatic lung tissues by real-time RT-PCR (Fig 5). Consistent with the ELISA results, treatment with picroside II suppressed mRNA expressions of *Il4*, *Il13*, *Il33*, *Mcp1*, and *Muc5ac*. The expression of *Il5* mRNA was also suppressed by picroside II, but to a lesser extent. The mRNA level of *Ifng* was significantly increased by picroside II. On the contrary, the level of *Ifng* mRNA was not significantly changed by dexamethasone. Recent evidence suggests that the IL-17 may also have a role in several asthma models [30]. However, we found that there was no meaningful decrease in the levels of *Il17* mRNA by picroside II treatment. Taken together, these results suggest that picroside II may control the inflammatory cytokines and mediators through transcriptional mechanisms.

### Effects of picroside II on transcription factors

**Fig 6:**
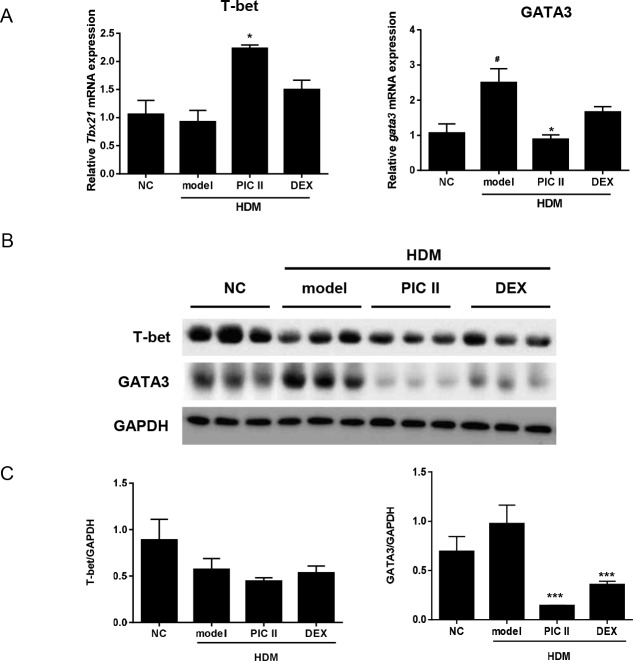
Effects of picroside II on the expression of transcription factors, T-bet and GATA3 in lung tissues. (A) T-bet and GATA3 mRNA expression were determined by real-time RT-PCR. The data were normalized to *Gapdh* gene expression. (B) T-bet and GATA3 protein were analyzed by western blot. (C) The western blot was quantitated by ImageJ. The levels of T-bet and GATA3 were calculated over GAPDH. NC; normal control mice treated with saline only, model; HDM-sensitized/challenged mice, PIC II; picroside II (30㎎/㎏) + HDM-sensitized/challenged mice, DEX; dexamethasone + HDM-sensitized/challenged mice. All data are representative of three independent experiments and represented as the mean ± SEM (n = 6mice/group). ^#^p<0.05, compared with normal control (NC); *p<0.05 and ***p<0.001, compared with model group.

T-bet and GATA3 are master transcription factors governing the development of native CD4+ T cells to Th1 and Th2 cells, respectively. Differential expression of Th1 and Th2 cytokines by picroside II led us to examine the mRNA and protein expressions of T-bet and GATA3 in lung tissues. Results from real-time RT-PCR revealed that picroside II significantly upregulated the expression of *Tbx21* (encoding T-bet) mRNA, but downregulated the expression of *Gata3* mRNA. Dexamethasone-treated mice showed no statistically significant differences compared with model mice in *Tbx21* and *Gata3* expressions (Fig 6A).

Western blot revealed that the expression of GATA3 protein was significantly reduced by picroside II compared with the model mice. The extent of inhibition by picroside II was even greater than that by dexamethasone. However, picroside II did not change the expression of T-bet (Fig 6B and 6C). These results suggest that the suppression of Th2 cytokines by picroside II may be mediated by a reduction in GATA3 expression.

### Effects of picroside II on Th2 cell differentiation

**Fig 7:**
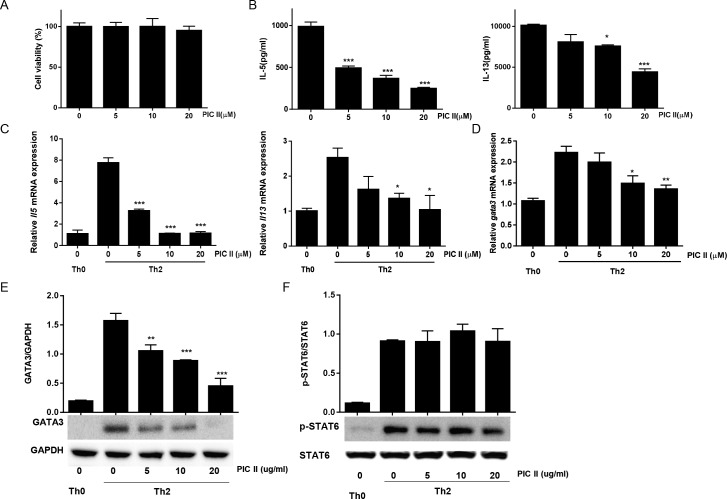
Effects of picroside II on mRNA levels and protein expression of Th2-related cytokines and transcription factor in developing Th2 cells. (A) Cytotoxicity of picroside II was assessed by CCK-8 assay. (B) The cytokine levels of IL-5 and IL-13 were measured using ELISA. (C) Th2-related cytokines (*Il5*, and *Il13)* and (D) *gata3* were determined from the activated Th2 cells by real-time RT-PCR. (E) Western blotting of GATA3 and (F) STAT6 phosphorylation were analyzed from the activated Th2 cells. Each group was quantitated by ImageJ, the levels of GATA3 and p-STAT6 were calculated over GAPDH and STAT6, respectively. Data are presented as mean ± SEM of each group. *p<0.05, **p<0.01, and ***p<0.001 indicate statistically significant difference compared with the control (Th2 cells, alone).

We next asked whether picroside II directly inhibits differentiation of Th2 cells. We isolated CD4+ T cells and stimulated with anti-CD3 and anti-CD28 in the presence of IL-4 and anti-IFNγ for Th2 differentiation. When we measured cell viability after treatment of picroside II (5, 10, and 20 μM) in CD4+ T cells, picroside II showed no cytotoxicity up to 20μM (Fig 7A). Thus, we applied picroside II at concentrations less than 20μM in the subsequent experiments. Addition of picroside II in developing Th2 cells decreased the levels of IL-5 and IL-13, in a dose dependent manner (Fig 7B). To confirm the effect of picroside II on the cytokine production at a single-cell level, we performed flow cytometric analyses. As expected, IL-13-secreting cells were gradually decreased with the increasing dose of picroside II (data not shown). Next, the gene transcript levels of *Il5* and *Il13* were evaluated by real-time RT-PCR. Consistent with the decreased protein levels of IL-5 and IL-13, the gene transcript levels of *Il5* and *Il13* were significantly inhibited by picroside II (Fig 6C).

We further investigated whether picroside II could reduce GATA3 level in developing Th2 cells as seen *in vivo*. We confirmed that picroside II dose-dependently suppressed the expression of GATA3 mRNA (Fig 6D) and protein (Fig 6E). IL-4 dependent STAT6 phosphorylation has been reported to play a critical role during Th2 differentiation [31]. Thus, we tested whether picroside II influences STAT6 signaling. However, western blot revealed that picroside II did not affect STAT6 phosphorylation in developing Th2 cells (Fig 6F).

## Discussion

Allergic asthma is a heterogeneous disorder with different phenotypes caused by various factors. So far, a drug that can completely cure asthma has not yet been developed. Although dexamethasone, a type of corticosteroids, is used for acute exacerbations of asthma, there are multiple adverse effects of this drug including adrenal and growth suppression [32]. Thus, herbal medicine could be attractive alternative therapies that have relatively low toxicity to treat asthma. Picroside II is one of constituents of YPL-001, which is being developed as a botanical drug of COPD treatment. Recently, we reported that verproside is a major biologically active compound of this plant, showing potent anti-inflammatory, antioxidant, and anti-asthmatic activities [33, 34]. However, verproside is predominantly metabolized to picroside II *in vivo* [35]. These information led us to evaluate the protective effects of picroside II on airway inflammation. Initially, when we examined the inhibitory effects of YPL-001, verproside, and picroside II on Th2 cytokine secretion, picroside II exhibited the most potent inhibitory activity (data not shown). Next, we have compared the extent of inhibition by picroside II and YPL-001 on allergic airway inflammation by analyzing the inflammatory cells in BALF of the HDM-treated mice. The results indicated that picroside II treatment reduced the inflammatory cells to a greater extent than YPL-001. Moreover, the efficacy of picroside II is comparable with that of the dexamethasone. For these reasons, we focused on the protective effect of picroside II on allergic asthma in the present study.

It is generally believed that allergic asthma is primarily mediated by Th2 cells, and associated with imbalance of Th1/Th2 cells [36]. Th2 cells produce high level of IL-4, IL-5 and IL-13 which are closely associated with asthma symptoms. IL-4 preferentially regulates IgE synthesis, IL-5 activates eosinophils while IL-13 increases mucus production. In contrast, IFN-γ is one of the main Th1-related cytokines, known to restrain the progress of asthma. Our ELISA results showed that picroside II markedly inhibited Th2 cytokines (inducing IL-4, IL-5 and IL-13), whereas elevated levels of Th1 cytokine, IFN-γ in BALF. In line with cytokine profiles, picroside II inhibited total IgE, HDM-specific IgE and HDM-specific IgG1 in serum, and inflammatory cell infiltration and mucus hypersecretion in lung tissues. We further demonstrate that picroside II inhibited Th2 cytokines at the transcription levels of the genes in lung tissues. Similar to ELISA results, mRNA levels of *Ifng* were significantly increased by picroside II. However, unlike picroside II, dexamethasone did not significantly suppress *Ifng* mRNA. These findings suggest that picroside II may confer clinical benefits over corticosteroids. It is important to note that picroside II also ameliorated HDM-induced increases of *Mcp1*, *Muc5ac and Il33* mRNA levels. Actually, MCP1 and MUC5AC are responsible for inflammatory cells infiltration[37] and hypersecretion of mucus [38]. Indeed, lung histological analysis revealed reduced inflammatory cells and attenuated mucus hypersecretion. Accumulating evidence also indicate that IL-33 is implicated in various form of allergic asthma [39].

T-bet and GATA3 are master regulators for Th1 and Th2 differentiation of CD4+ T cells, respectively [14, 15]. Accordingly, the expression of T-bet and GATA3 were detected by real-time RT-PCR and western blot in HDM-treated mice. Indeed, picroside II selectively inhibited the mRNA and protein levels of GATA3, but increased the level of *Tbx21* mRNA encoding T-bet. These data are consistent with Th1/Th2 cytokine analysis. Thus, the mechanisms of picroside II on airway inflammation may be mediated by suppressing GATA3 and concomitant Th2-type cytokine gene expression.

We further confirmed that the anti-inflammatory effects of picroside II using splenic CD4+ T cells. Consistent with *in vivo* studies, picroside II suppressed the secretion of Th2 cytokines, IL-5 and IL-13, and the expression of GATA3 at both the mRNA and protein levels. On the other hand, our *in vivo* studies showed that picroside II did not significantly affect the expression of *Il17* mRNA. We further confirmed this using *in vitro* Th17 differentiation studies that also showed no suppressive effect of picroside II on the IL-17 secretion (data not shown). Therefore, we conclude that picroside II exerts its suppressive effects on HDM-induced asthma through inhibition of Th2, rather than the Th17 response. The STAT6 signaling pathway is mainly induced by IL-4, which induces the expression of GATA3 [31, 40]. Therefore, we examined whether GATA3 inhibition by picroside II is associated with STAT6 signaling by western blot. However, we failed to detect the inhibitory effects of picroside II on STAT6 phosphorylation in developing Th2 cells. In fact, GATA3 expression can also be induced by T cell receptor (TCR) signaling [41] and other pathways, such as Notch [42] and WNT signaling [43]. Further studies will be necessary to elucidate the molecular mechanism for the inhibition of GATA3 by picroside II.

In summary, we demonstrated that picroside II suppressed airway inflammation in HDM-induced asthmatic mice. We further demonstrated that picroside II inhibited the Th2 cytokines via inhibiting the transcription factor GATA3 in HDM-induced asthmatic mice as well as developing Th2 cells. Taken together, our findings suggest that picroside II may have therapeutic potential as an alternatives to corticosteroids for the treatment of allergic asthma.

## Supporting Information

S1 Fig ^1^H, ^13^C-NMR data. (A) Spectra 1. ^1^H NMR spectrum of picroside II by using 400 MHz. (B) Spectra 2. ^13^C NMR spectrum of picroside II by using 100 MHz. light brownish powder; mp 137–140°C; HRESIMS *m/z* 511.1436 [M‒H]^‒^ (calcd for C_23_H_17_O_13_, 511.1452); [α]_D_^20^–158.7° (c 0.11, CH_3_OH); ^1^H-NMR (400 MHz, DMSO-*d*_*6*_) *δ*2.47 (1H, d, *J* = 9.3 Hz, H-9), 2.58 (1H, dd, *J* = 9.3, 7.6 Hz, H-5), 3.03 (1H, m, Glc-4''), 3.07 (1H, dd, *J* = 8.0, 7.6 Hz, Glc-2''), 3.14 (1H, m, H- Glc-5''), 3.18 (1H, m, Glc-3''), 3.44 (1H, dd, *J* = 13.2, 6.4 Hz, Glc-6''), 3.72 (1H, d, *J* = 13.2 Hz, Glc-6''), 3.67 (1H, br s, H-7), 3.72 (1H, m, H-10), 3.93 (1H, d, *J* = 13.2 Hz, H-10), 3.82 (3'-OCH_3_), 4.63 (1H, d, *J* = 7.6 Hz, Glc-1''), 4.97 (1H, dd, *J* = 5.8, 4.4 Hz, H-4), 5.06 (1H, d, *J* = 7.6 Hz, H-6), 5.11 (1H, d, *J* = 9.3 Hz, H-1), 6.42 (1H, d, *J* = 5.8 Hz, H-3), 6.85 (1H, d, *J* = 7.6 Hz, H-5'), 7.46 (1H, s, H-2'), 7.52 (1H, d, *J* = 7.6 Hz, H-6'); ^13^C NMR (100 MHz) *δ*35.2 (C-5), 41.8 (C-9), 55.7 (3'-OCH_3_), 58.2 (C-7), 58.5 (C-10), 61.4 (Glc-6''), 65.9 (C-8), 70.3 (Glc-4''), 73.5 (Glc-2''), 76.5 (Glc-3''), 77.5 (Glc-5''), 79.4 (C-6), 93.0 (C-1), 97.9 (Glc-1''), 101.9 (C-4), 112.7 (C-2'), 115.3 (C-5'), 119.9 (C-1'), 123.9 (C-6'), 141.1 (C-3), 147.5 (C-3'), 152.1 (C-4'), 165.6 (C-7').
(TIF)

S2 Fig UPLC-PDA-QTOF-MS analysis. (A) UPLC-PDA chromatogram of isolated picroside II. The ethanol extracts and subfractions were analyzed by UPLC PDA QTOF-MS. Chromatographic separations were performed on a 2.1 × 100 mm, 1.7 μm ACQUITY BEH C18 chromatography column. The column temperature was maintained at 35°C, and the mobile phases A and B were water with 0.1% formic acid and acetonitrile with 0.1% formic acid, respectively. The gradient duration program was: 0–1 min, 10% B; 1–10.5 min, 10–23% B; 10.5–12.0 min, 23–98% B; wash to 15.0 min with 98% B; and a 1.5 min recycle time. The flow rate was 0.4 mL/min. (B) UPLC-QTOF-MS and HREIMS data of picroside II. The mass spectrometer was operated in positive ion mode. N_2_ was used as the desolvation gas. The desolvation temperature was set to 350°C at a flow rate of 500 L/h and source temperature of 100°C. The capillary and cone voltages were set to 2300 and 35 V, respectively. The Q-TOF premier^TM^ was operated in V mode with 9000 mass resolving power. The data were collected for each test sample from 100 to 1500 Da with a 0.25 s scan time and a 0.01 s interscan delay over a 15 min analysis time. Leucine-enkephalin was used as the reference compound (*m/z* 554.2615 in the negative mode) and an infusion flow rate of 1 μl/min.
(TIF)
