# Correction: Picroside II Attenuates Airway Inflammation by Downregulating the Transcription Factor GATA3 and Th2-Related Cytokines in a Mouse Model of HDM-Induced Allergic Asthma

**DOI:** 10.1371/journal.pone.0170832

**Published:** 2017-01-20

**Authors:** Jin Choi, Bo Kyong Choi, Jin seok Kim, Jae-Won Lee, Hyun Ah Park, Hyung Won Ryu, Su Ui Lee, Kwang Woo Hwang, Won-Kee Yun, Hyoung-Chin Kim, Kyung-Seop Ahn, Sei-Ryang Oh, Hyun-Jun Lee

There are errors in the Funding section. The correct funding information is as follows: This work was supported by grants from the Korea Research Institute of Bioscience and Biotechnology Research Initiative Program (KGM1221622) and Center for Women In Science Engineering and Technology (WISET2016-167). The funders had no role in study design, data collection and analysis, decision to publish, or preparation of the manuscript.
