# Supplementary figures and images for: Picroside II Attenuates Airway Inflammation by Downregulating the Transcription Factor GATA3 and Th2-Related Cytokines in a Mouse Model of HDM-Induced Allergic Asthma
Source: PLoS One. 2016 Nov 21;11(11):e0167098. doi: 10.1371/journal.pone.0167098 (PMC5117775; doi:10.1371/journal.pone.0167098)

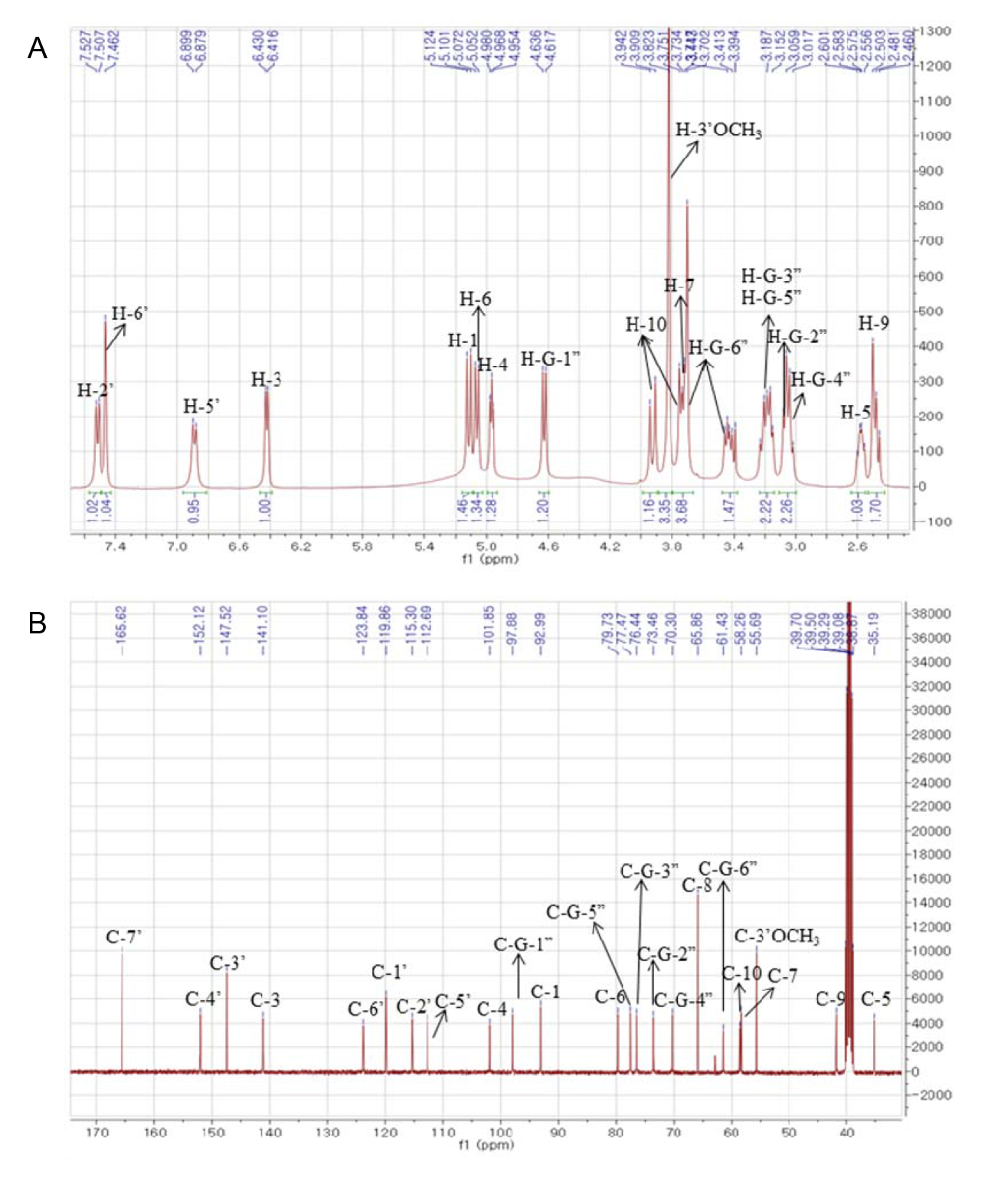

Supplement: S1 Fig — (A) Spectra 1. 1H NMR spectrum of picroside II by using 400 MHz. (B) Spectra 2. 13C NMR spectrum of picroside II by using 100 MHz. light brownish powder; mp 137–140°C; HRESIMS m/z 511.1436 [M‒H]‒ (calcd for C23H17O13, 511.1452); [α]D20–158.7° (c 0.11, CH3OH); 1H-NMR (400 MHz, DMSO-d6) δ2.47 (1H, d, J = 9.3 Hz, H-9), 2.58 (1H, dd, J = 9.3, 7.6 Hz, H-5), 3.03 (1H, m, Glc-4''), 3.07 (1H, dd, J = 8.0, 7.6 Hz, Glc-2''), 3.14 (1H, m, H- Glc-5''), 3.18 (1H, m, Glc-3''), 3.44 (1H, dd, J = 13.2, 6.4 Hz, Glc-6''), 3.72 (1H, d, J = 13.2 Hz, Glc-6''), 3.67 (1H, br s, H-7), 3.72 (1H, m, H-10), 3.93 (1H, d, J = 13.2 Hz, H-10), 3.82 (3'-OCH3), 4.63 (1H, d, J = 7.6 Hz, Glc-1''), 4.97 (1H, dd, J = 5.8, 4.4 Hz, H-4), 5.06 (1H, d, J = 7.6 Hz, H-6), 5.11 (1H, d, J = 9.3 Hz, H-1), 6.42 (1H, d, J = 5.8 Hz, H-3), 6.85 (1H, d, J = 7.6 Hz, H-5'), 7.46 (1H, s, H-2'), 7.52 (1H, d, J = 7.6 Hz, H-6'); 13C NMR (100 MHz) δ35.2 (C-5), 41.8 (C-9), 55.7 (3'-OCH3), 58.2 (C-7), 58.5 (C-10), 61.4 (Glc-6''), 65.9 (C-8), 70.3 (Glc-4''), 73.5 (Glc-2''), 76.5 (Glc-3''), 77.5 (Glc-5''), 79.4 (C-6), 93.0 (C-1), 97.9 (Glc-1''), 101.9 (C-4), 112.7 (C-2'), 115.3 (C-5'), 119.9 (C-1'), 123.9 (C-6'), 141.1 (C-3), 147.5 (C-3'), 152.1 (C-4'), 165.6 (C-7'). (TIF) [file pone.0167098.s001.tif]

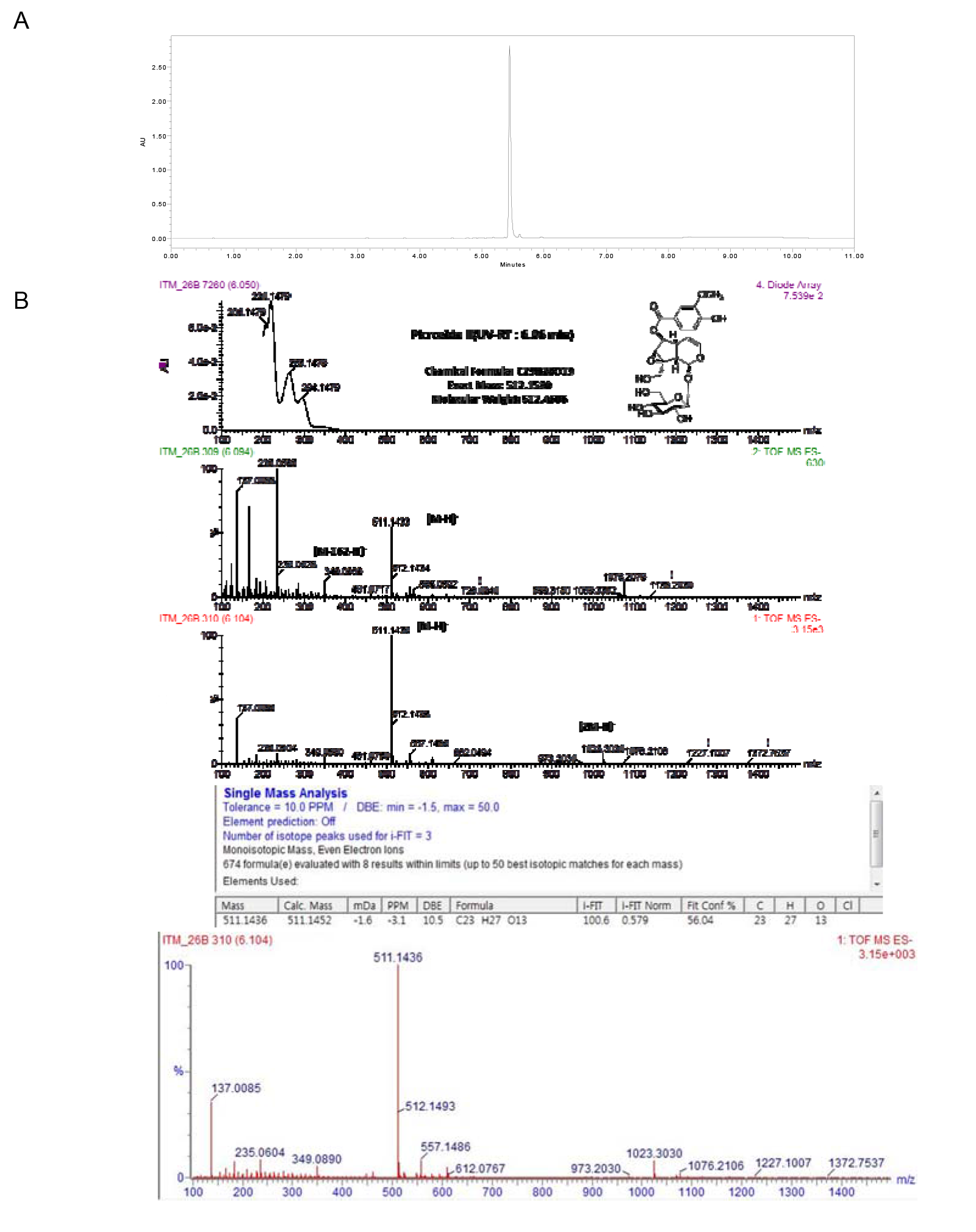

Supplement: S2 Fig — (A) UPLC-PDA chromatogram of isolated picroside II. The ethanol extracts and subfractions were analyzed by UPLC PDA QTOF-MS. Chromatographic separations were performed on a 2.1 × 100 mm, 1.7 μm ACQUITY BEH C18 chromatography column. The column temperature was maintained at 35°C, and the mobile phases A and B were water with 0.1% formic acid and acetonitrile with 0.1% formic acid, respectively. The gradient duration program was: 0–1 min, 10% B; 1–10.5 min, 10–23% B; 10.5–12.0 min, 23–98% B; wash to 15.0 min with 98% B; and a 1.5 min recycle time. The flow rate was 0.4 mL/min. (B) UPLC-QTOF-MS and HREIMS data of picroside II. The mass spectrometer was operated in positive ion mode. N2 was used as the desolvation gas. The desolvation temperature was set to 350°C at a flow rate of 500 L/h and source temperature of 100°C. The capillary and cone voltages were set to 2300 and 35 V, respectively. The Q-TOF premierTM was operated in V mode with 9000 mass resolving power. The data were collected for each test sample from 100 to 1500 Da with a 0.25 s scan time and a 0.01 s interscan delay over a 15 min analysis time. Leucine-enkephalin was used as the reference compound (m/z 554.2615 in the negative mode) and an infusion flow rate of 1 μl/min. (TIF) [file pone.0167098.s002.tif]
